# Supplementary material for: Modulation of the Nernst Thermoelectrics by Regulating the Anomalous Hall and Nernst Angles
Source: Adv Sci (Weinh). 2024 Nov 21;12(2):2411702. doi: 10.1002/advs.202411702 (PMC11727403; doi:10.1002/advs.202411702)
Supplement: Supplementary file 1 — Supporting Information [file ADVS-12-2411702-s001.docx]

Supplementary Information


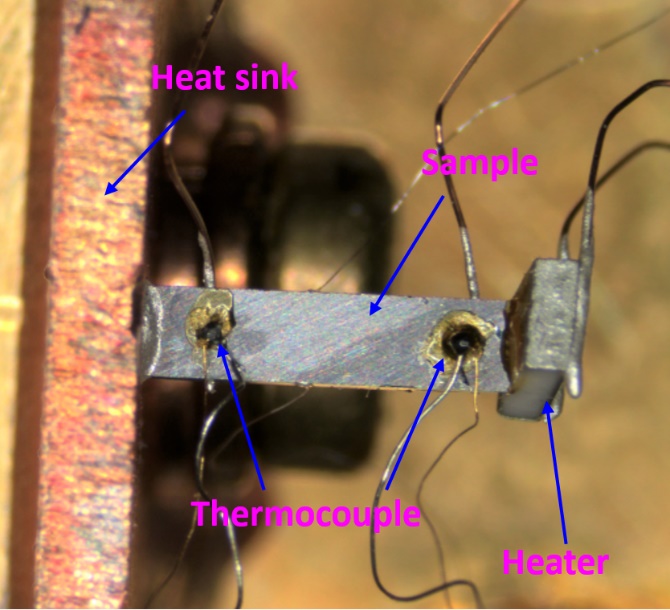
**1. The configuration image of thermoelectric measurements**

**Figure S1.** The configuration image of thermoelectric measurements. The used sample was cut and polished to a cuboid. The resistance chip heater located on the right-hand side provides a heat flow to the sample, and the copper on the left, anchored to the base, acts as a heat sink for establishing a stable temperature difference$\Delta T$. This temperature difference is monitored by thin chromel-AuFe_0.07%_ thermocouples (*φ* = 25 μm).


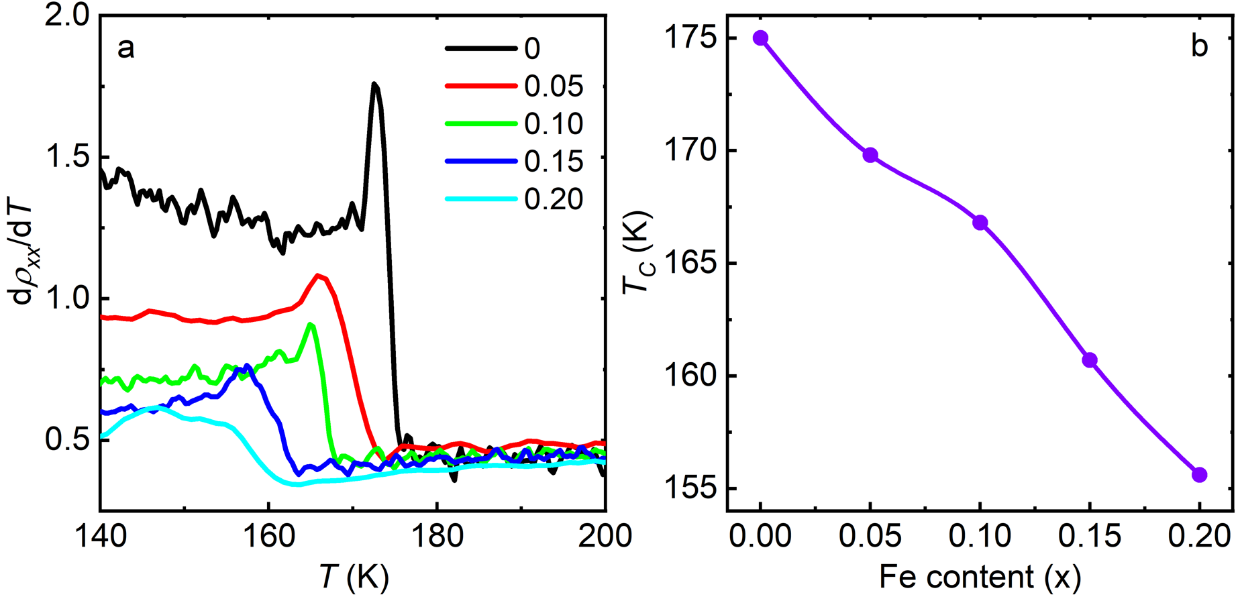
**2. *T_C_* characterization by *dρ/dT* plots**

**Figure S2.** a) The temperature dependent *dρ_xx_/dT*. b) The evolution of Curie temperature *T_C_* with different Fe doping characterized by *dρ_xx_/dT*, which is consistent with the former work of the *dρ/dT* plot, indicating the measured crystals are reliable. Noted *T_C_* here is slightly higher than Ref. [1] because of different characterization methods to determine Curie temperature, the latter is characterized by *M(T)* curves.


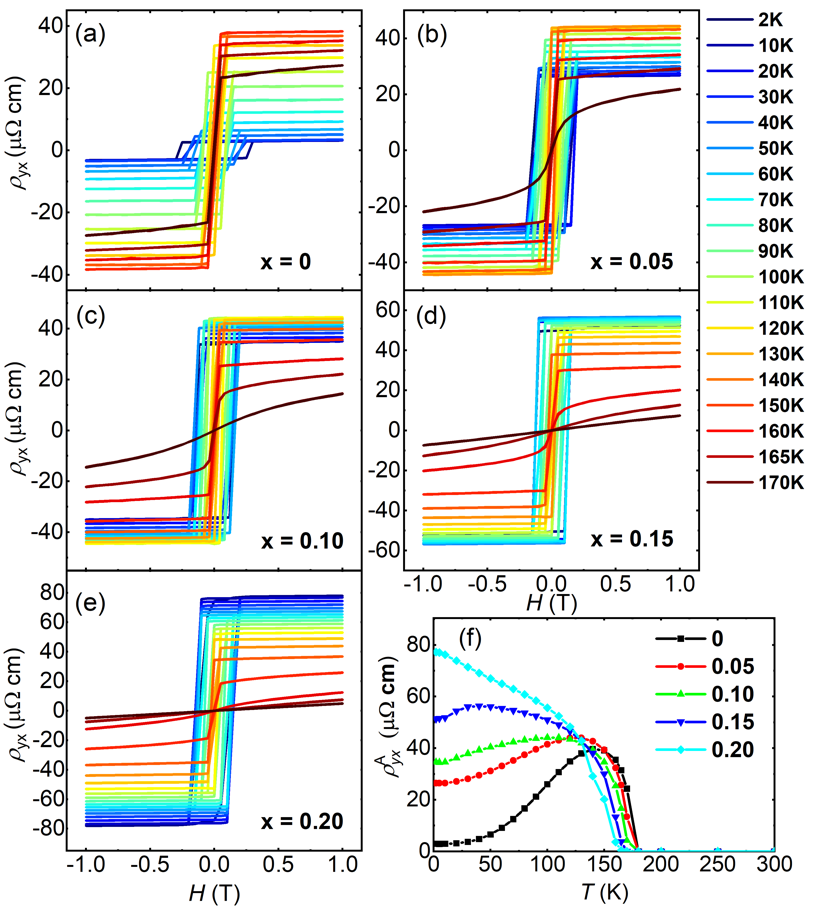
**3. Detailed field dependent Hall resistivity *ρ_yx_***

**Figure S3.** a-e) Detailed magnetic field dependence of Hall Effect for different Fe doping content with *I* // a and *H* // c, which further yields temperature-dependent anomalous Hall resistivity $\text{ρ}_{\text{yx}}^{\text{A}}$ shown in (f.


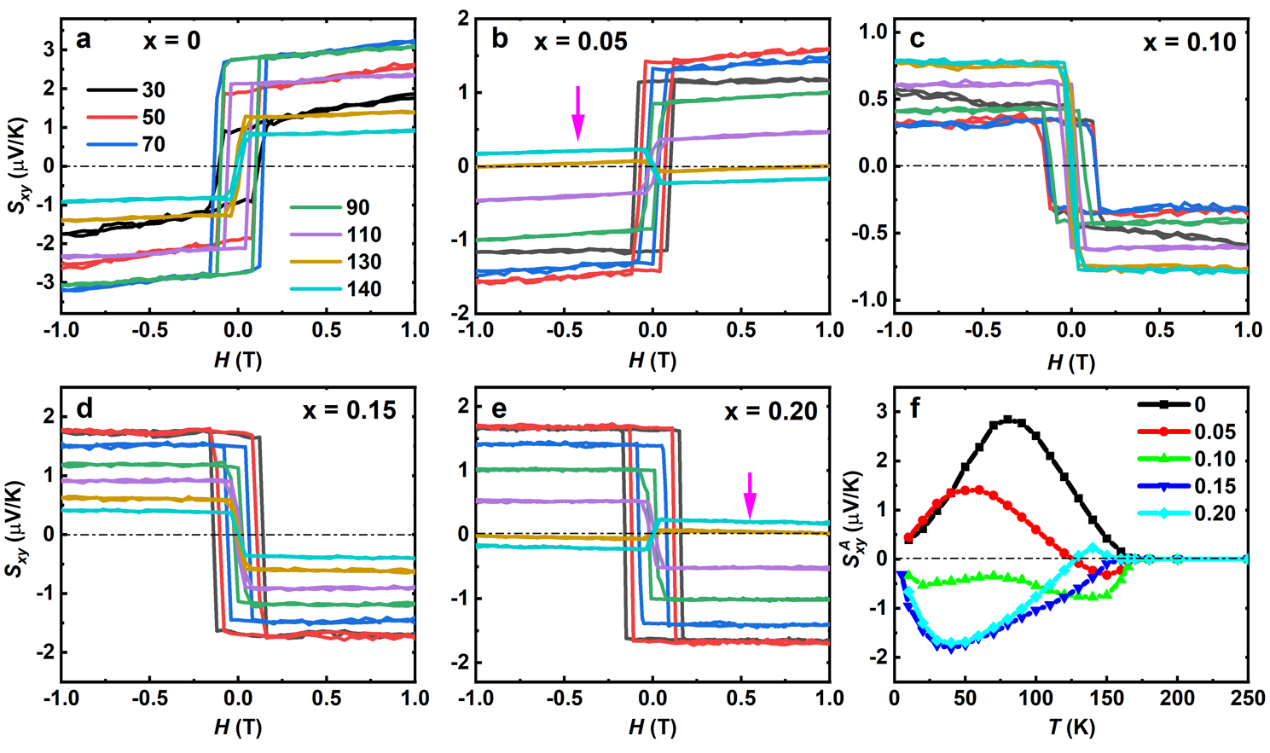
**4. Detailed field dependent Nernst signal *S_yx_***

**Figure S4.** Anomalous Nernst effect of Co_3-_*_x_*Fe*_x_*Sn_2_S_2_. a-e) The Nernst effect *S_xy_* as a function of magnetic field at selected temperatures with *x* = 0, 0.05, 0.10, 0.15 and 0.20 successively. In these figures, one can clearly observe that the anomalous Nernst effect $\text{S}_{\text{xy}}^{\text{ A}}$changes the sign from positive to negative as Fe content increases. More specifically, $\text{S}_{\text{xy}}^{\text{ A}}$ also changes the sign at *x* = 0.05 and 0.20 with increasing temperature. f) Summarized $\text{S}_{\text{xy}}^{\text{ A}}$as a function of temperature.

**5. TYJ model analysis of AHC**

To further verify the reliability of the measured samples, we also did the TYJ model [2] estimation to AHC. According to the TYJ model$\sigma_{xy}^{A}=-a\sigma_{xx0}^{-1}\sigma_{xx}^{2}-b$, $\sigma_{xy}^{A}$ is expected to be linear to $\sigma_{xx}^{2}$, and the intrinsic AHC can be estimated as a constant b. As shown in Figure S4, AHC of Co_3-x_Fe_x_Sn_2_S_2_ at low temperature can be linearly extrapolated to zero, then yields the intrinsic $\sigma_{xy}^{Int.}$ (see in the inset). By subtracting the intrinsic contribution, the extrinsic part of AHC $\sigma_{xy}^{Ext.}$ was obtained (see in the main text of Figure 4a), which is gradually increasing as Fe doped into, and dominate the total AHC when x ≥ 0.1. The separation results are consistent with previous work, further affirming the reliable nature of the measured samples. It is noted that the zero field electric resistivity *ρ_xx_* (x = 0.15) here is different from it in Ref. [1]. *ρ_xx_* (x = 0.15) in this manuscript is absence of Kondo effect at low temperature, thus the corresponding AHC could also be separated by TYJ model.


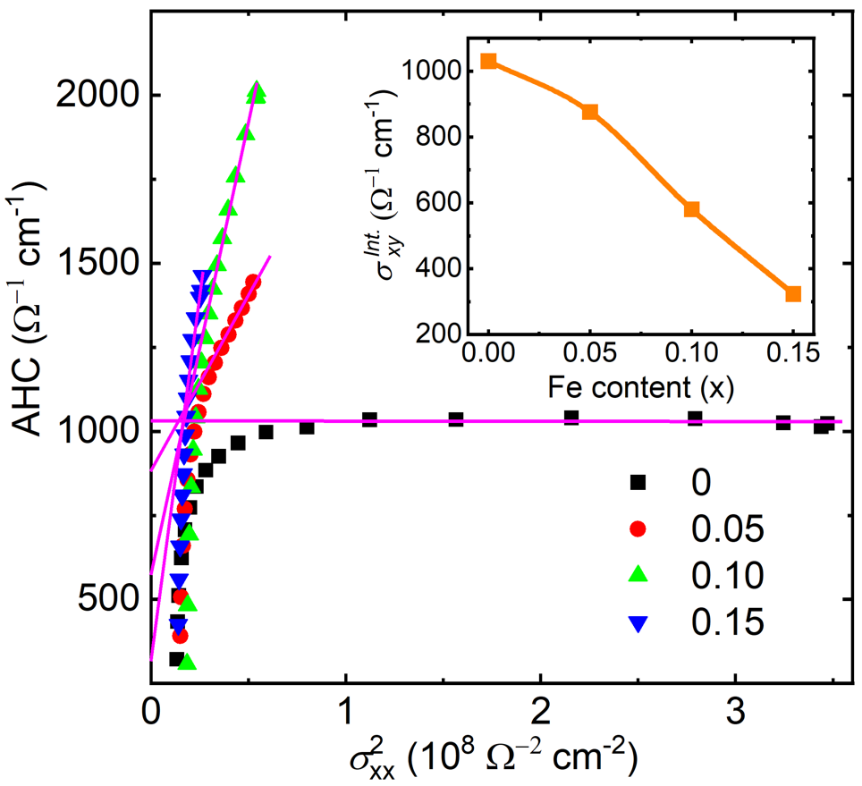


**Figure S5**. TYJ model fitting of AHC. The solid green lines represent the linear dependence of $\sigma_{xx}^{2}$, which are extended to zero yields the intrinsic contribution $\sigma_{xy}^{Int.}$. Thus, the Fe content dependence of $\sigma_{xy}^{Int.}$ is shown in the inset.

**6. Theoretical calculations on the intrinsic AHC and ANC**

First-principles calculations based on the density-functional theory (DFT) were performed using the projector-augmented wave (PAW) method as implemented in the Vienna Ab Initio Simulation (VASP) Package.^[2, 3]^ The generalized gradient approximation (GGA) of Perdew-Burke-Ernzerhof (PBE) for the exchange-correlation functional was used.^[4]^ The cut-off energy of plane wave basis was set 500 eV and the first Brillouin zone of the reciprocal space was sampled with k-point meshes of 15×15×15 for Co_3_Sn_2_S_2_. The structures were fully relaxed until the force on each atom is smaller than 0.001 eV/Å. By projecting the Bloch wavefunctions into Wannier functions,^[5]^ the tight-binding model Hamiltonian based on Wannier functions was constructed. The relationship between the anomalous Hall conductivity and Berry curvature were calculated by the Kubo formula approach.^[6]^ Then the energy-dependent AHC was obtained by using WannierBerri code,^[7]^ as shown in Figure S5. According to the Mott relation, the expression of the anomalous off-diagonal thermoelectric coefficient is $\alpha_{xy}^{A}\left( T,\mu\right)=-\frac{1}{e}\int d\epsilon\frac{\partial f\left( \epsilon-\mu,T \right)}{\partial\epsilon}\frac{\epsilon-\mu}{T}\sigma_{xy}^{A}(\epsilon)$,^[8]^ where$\sigma_{xy}^{A}(\epsilon)$ is the chemical-potential-dependent anomalous Hall conductivity at the zero temperature, $f\left( \epsilon-\mu,T \right)$ is the Fermi-Dirac distribution function, and $\mu$ is the chemical potential. We have calculated $\sigma_{xy}^{A}$ based on the Berry curvature extracted from the ab initio **
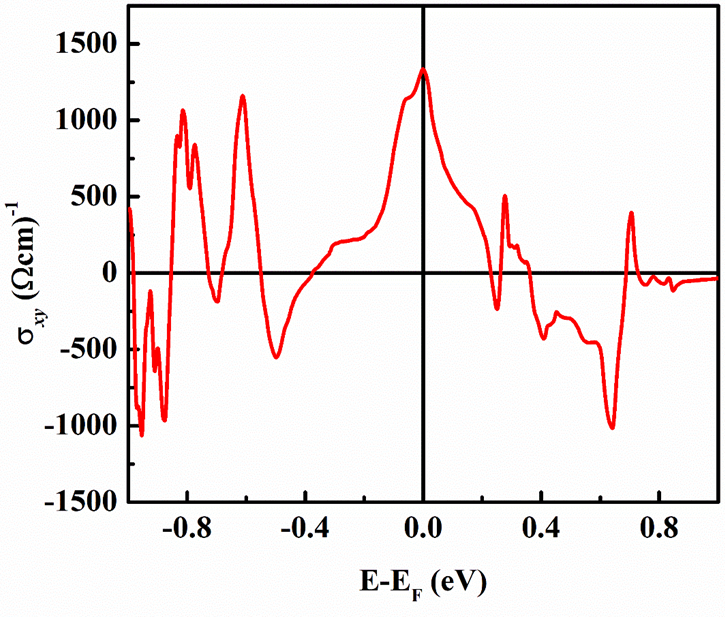
**calculations and future obtained $\alpha_{xy}^{A}$.

**Figure S6**. Energy dependence of the anomalous Hall conductivity of Co_3_Sn_2_S_2_.

**7. The longitudinal and transverse thermoelectric conductivity**

In the zero field, the anomalous behavior of the Hall and Nernst effects can be written as the following tensor formula

$\alpha=\sigma S$ (S1)

Where $\alpha$ is the Peltier tensor, describing longitudinal and transverse thermoelectric conductivities. $\sigma\mathrm{and} S$ are the electrical conductivity tensor and the thermoelectric tensor, the former describing longitudinal and Hall conductivities and the latter Seebeck and Nernst signals. Furthermore, the electrical conductivity can be expressed as the inverse of the electrical resistivity tensor,

$\sigma=\rho^{-1}$ (S2)

Here, we focus on the xy plane

$$\alpha=\left( \begin{matrix} \alpha_{xx} & \alpha_{xy} \\ \alpha_{yx} & \alpha_{yy} \end{matrix} \right)=\frac{1}{det\rho}\left( \begin{matrix} \rho_{yy} & {-\rho}_{xy} \\ {-\rho}_{yx} & \rho_{xx} \end{matrix} \right)\left( \begin{matrix} S_{xx} & S_{xy} \\ S_{yx} & S_{yy} \end{matrix} \right)$$

$=\frac{1}{\rho_{xx}\rho_{yy}-\rho_{xy}\rho_{yx}} \left( \begin{matrix} \rho_{yy}S_{xx}{- \rho}_{xy}S_{yx} & \rho_{yy}S_{xy}{- \rho}_{xy}S_{yy} \\ \rho_{xx}S_{yx}{- \rho}_{yx}S_{xx} & \rho_{xx}S_{yy}{- \rho}_{yx}S_{xy} \end{matrix} \right)$ (S3)

With $\rho_{xx}{=\rho}_{yy}$and $\rho_{xy}{=-\rho}_{yx}$ in Co_3_Sn_2_S_2_, $\alpha_{xx}$ and $\alpha_{yx}$ can be expressed as

$\alpha_{xx}=\frac{\rho_{yy}S_{xx}{- \rho}_{xy}S_{yx}}{\rho_{xx}\rho_{yy}-\rho_{xy}\rho_{yx}}=\sigma_{xx}S_{xx}{+ \sigma}_{yx}S_{yx}$ (S4)

$\alpha_{yx}=\frac{\rho_{xx}S_{yx}{- \rho}_{yx}S_{xx}}{\rho_{xx}\rho_{yy}-\rho_{xy}\rho_{yx}}=\sigma_{yx}S_{xx}{+ \sigma}_{xx}S_{yx}$ (S5)

Deviding each side of Eq. (S5) by $\sigma_{xx}S_{xx}$, we have

$\alpha_{yx}=\sigma_{xx}S_{xx}\left( \tan\theta_{H}+\tan\theta_{N} \right)$ (S6)

where $\tan\theta_{H}$is the anomalous Hall angle$\equiv\sigma_{yx}/\sigma_{xx}$, and $\tan\theta_{N}$is the anomalous Nernst angle$\equiv S_{yx}/S_{xx}$. In general, $\sigma_{xx}S_{xx}{\gg\sigma}_{yx}S_{yx}$ in Eq. (S4), so Eq. (S6) can be simplified as

$\alpha_{yx}=\alpha_{xx}\left( \tan\theta_{H}+\tan\theta_{N} \right)$ (S7)

It is important to note that if $\sigma_{yx}S_{yx}$ is not significantly smaller than $\sigma_{xx}S_{xx}$ in a given material, the formula (S7) will revert to (S6).

**References**

[1] J. Shen, Q. Zeng, S. Zhang, H. Sun, Q. Yao, X. Xi, W. Wang, G. Wu, B. Shen, Q. Liu, E. Liu, Advanced Functional Materials **2020**, 30, 202000830.

[2] P. E. Blochl, Phys Rev B Condens Matter **1994**, 50, 17953.

[3] G. Kresse, J. Furthmüller, Computational materials science **1996**, 6(1), 15-50.

[4] J. P. Perdew, K. Burke , M. & Ernzerhof, Physical review letters **1996**. 77(18), 3865.

[5] A. A. Mostofi, J. R. Yates, Y.-S. Lee, I. Souza, D. Vanderbilt, N. Marzari, Computer Physics Communications **2008**, 178, 685.

[6] D. Xiao, M.-C. Chang, Q. Niu, Reviews of Modern Physics **2010**, 82, 1959.

[7] S. S. Tsirkin, npj Computational Materials **2021**, 7, 33.

[8] H. Sawahata, N. Yamaguchi, S. Minami, F. Ishii, Physical Review B **2023**, 107, 024404.
